# A Spectrophotometric Method to Determine Minimal Erythema Dose for Ultraviolet Radiation in Human Skin

**DOI:** 10.3390/biomedicines12112544

**Published:** 2024-11-07

**Authors:** Eun Ju Lee, Chung Han Lee, Ji Hwoon Baek, Jae Sook Koh, Yong Chool Boo

**Affiliations:** 1 Skin Research Center, Dermapro Ltd., Seoul 06570, Republic of Korea; pololi-3@hanmail.net (E.J.L.); ohplaza@naver.com (C.H.L.); jhun100@nate.com (J.H.B.); 2 Department of Molecular Medicine, School of Medicine, Kyungpook National University, Daegu 41944, Republic of Korea; 3 BK21 Plus KNU Biomedical Convergence Program, Department of Biomedical Science, The Graduate School, Kyungpook National University, Daegu 41944, Republic of Korea; 4 Cell and Matrix Research Institute, Kyungpook National University, Daegu 41944, Republic of Korea

**Keywords:** minimal erythema dose, MED, ultraviolet radiation, UVR, erythema, redness, a* value, oxidative stress, spectrophotometer, instrumental evaluation

## Abstract

**Background/Objectives**: Ultraviolet radiation (UVR) induces oxidative stress in the skin by generating reactive oxygen species (ROS), which can lead to inflammatory conditions including erythema (a sign of sunburn). This clinical study aims to develop an instrumental evaluation method to determine the minimal erythema dose (MED) for UVR. **Methods**: Fourteen human subjects aged 27 to 57 years (48.93 ± 8.54) participated in this study. Six subsites were designated on the test skin site of each subject and irradiated with UVR at different doses. The examiner visually assessed erythema, determining the ‘visual MED’. Additionally, the a* value (a chrominance parameter presenting greenness to redness) was measured using a spectrophotometer as an indicator of erythema. The a* values of the UVR-irradiated subsites were compared to the non-irradiated control value, and the differences were referred to as Δa*. The Δa* value of the subsites irradiated with UVR at the ‘visual MED’ was referred to as the Δa*_VMED_ for each subject. The mean of the Δa*_VMED_ values of all subjects was chosen as a criterion value for the ‘instrumental MED’. The ‘instrumental MED’ was defined as the lowest dose of UVR that causes an Δa* value equivalent to the criterion value. The ‘visual MED’ and ‘instrumental MED’ values of all subjects were subjected to correlation analysis. **Results**: The mean of the Δa*_VMED_ values of all subjects was 1.88 ± 0.8. The means of the ‘visual MED’ and ‘instrumental MED’ values (in J m^−2^ unit) of all subjects were 300.14 ± 84.16 and 303.29 ± 77.99, respectively. In Pearson correlation analysis, the ‘instrumental MED’ and ‘visual MED’ values had a very strong positive correlation with each other (r = 0.864, *p* = 0.000). **Conclusions**: This study suggests that the instrumental evaluation method of MED based on the spectrophotometric measurement of the a* values can complement or replace the visual evaluation method and that this method will be useful in monitoring skin tolerance to oxidative stress affected by prooxidant factors and defensive factors.

## 1. Introduction

The skin is the outermost organ of our body and mediates interactions between various factors inside and outside the body [1]. In addition to its barrier function, the skin plays an important role in body temperature regulation, fluid secretion, gas exchange, and sensation, and it also protects against a wide range of external factors including ultraviolet radiation (UVR) [2]. Repeated exposure to high-intensity UVR induces oxidative stress in the skin by generating reactive oxygen species (ROS), which can lead to inflammation, aging, and cancer [3,4]. Various pigments in the skin, such as melanin, provide essential defense against it [5,6]. The primary skin color of humans is determined innately, and the ability to defend against UVR differs between races [7,8]. Conversely, UVR is an important factor in causing acquired changes in skin color as an adaptive response [9,10,11].

Since the skin is exposed to the outside, its changes in shape and color can be observed visually or instrumentally [12,13]. Erythema (reddening of the skin) is a perceptible sign of oxidative stress and inflammatory reactions observed in the skin [4,14]. The minimal erythema dose (MED) is the minimum dose of a stimulus (e.g., UVR) that causes erythema of the skin [15]. The MED varies depending on the individual’s skin color, gender, age, etc. [16]. The MED is also influenced by the presence and activity of antioxidants, such as vitamins C and E, which scavenge ROS generated by UVR and enhance skin tolerance to oxidative stress [17]. The MED for UVR is used to determine the sun protection factor (SPF) or UVR-blocking effects of cosmeceuticals and other commercial products [18]. Therefore, the MED can be widely used as an indicator of skin tolerance to oxidative stress affected by prooxidant factors (e.g., UVR, ROS) and defensive factors (e.g., sunscreen, antioxidants).

In determining the MED, skin erythema has been assessed visually by the examiners [15]. This visual evaluation method has limitations in maintaining the consistency of results because it relies on the subjective sensory observation of the examiner. The instrumental methods to evaluate the MED involving spectrometric measurements of skin color parameters have been used [15,19], but are not technically well established.

The skin color is expressed using the Commission Internationale de l’Eclairage Lab color space composed of L*, a*, and b* parameters [20,21]. L* is a luminance parameter presenting lightness and a* and b* are chrominance parameters presenting greenness to redness and blueness to yellowness, respectively. The skin color can be represented by the individual typology angle (ITA°) [22,23]. The higher the ITA° value, the lighter the skin color and the more the skin is sensitive to UVR. It is possible to approximately predict the MED for UVR from the ITA° value of each subject’s skin determined before UVR exposure [24].

Fitzpatrick’s classification of skin types based on sun reactivity divides them into six skin types from the most sensitive type I to the most insensitive type VI [25]. It was shown that the MED gradually increased and the L* value gradually decreased from skin types I to VI, and that there was a negative correlation between the MED and L* values [26]. However, no significant difference was observed between the MED values of Koreans with Fitzpatrick skin type III and type IV, and the skin color analysis after UVR irradiation at the MED showed no difference in the L* and a* values between these two skin types, even though the b* value was slightly smaller in type IV [27]. Thus, there are limitations in predicting the UVR reactivity based on Fitzpatrick skin types or skin color parameters measured at a specific time. It is assumed that the skin sensitivity to UVR may be related to the relative changes in skin color parameters affected by UVR exposure, however, no previous studies have examined this notion.

The primary goal of the present study is to develop an instrumental evaluation method of the MED for UVR that could complement or replace the existing visual evaluation method. In a clinical study of 14 human subjects, we examined an instrumental evaluation method of the MED involving the analysis of the differences in the skin color parameters measured with a spectrophotometer with and without UVR irradiation. The instrumental evaluation method of the MED for UVR gave results almost equivalent to those of the visual evaluation method. This novel method would find wide applications in monitoring skin inflammation due to the oxidative stress caused by UVR, chemicals, heat, and other stimuli.

## 2. Materials and Methods

### 2.1. Clinical Study and Human Subjects

The clinical study protocols No. 1-220777-A-N-01-DICN22204, 22241, and 22248 were approved in October and November 2022 by the Institutional Review Board of Dermapro Co., Ltd. (Seoul, Republic of Korea), and the human test proceeded from November to December 2022. The clinical study was conducted in accordance with ethical principles based on the Declaration of Helsinki [28].

The exclusion criteria for subjects were as follows: pregnant or breastfeeding, taking photosensitizing medications, taking anti-inflammatory medications, having systemic skin diseases (including dysplastic nevi), having a history of photoallergy or photosensitivity, tanning within 8 weeks of participating in the UVR test, exposure to UVR on the back within 8 weeks of participating in the UVR test, having scars or moles on the test site, having signs of sun damage on the test site, having excessive hair on the test site, having protrusions or curves due to the skeleton on the test site, etc.

Healthy male or female volunteers who did not meet the above exclusion criteria were recruited. The purpose and method of the clinical study, expected results, and potential adverse reactions were explained to them, and their willingness to participate was asked. Consent forms for participation in the test were obtained from those who consented.

The subjects visited the research center on the day of the test. After washing their test skin site, the subjects rested for 20 min and underwent UVR irradiation in a laboratory maintained at 22 ± 2 °C and 50 ± 5% relative humidity. Visual and instrumental evaluations of the UVR-irradiated skin site were performed during the next visit to the laboratory on the following day.

### 2.2. Spectrophotometric Measurement of Basic Skin Color Parameters

The skin color parameters of human subjects, such as L*, a*, and b*, were measured using a spectrophotometer CM-2500d (Minolta, Osaka, Japan) [21,29]. The measurement of skin color parameters L*, a*, and b* on the back skin site of each subject was repeated three times, and the data were averaged. The ITA° was calculated from the measured L* and b* values using the following equation: ITA° = [ArcTangent ((L* − 50)/b*)] × 180/3.14159 where ArcTangent is expressed in radians [22,23]. The ITA° value was used to calculate the ‘predicted MED’ of each subject [24].

### 2.3. UVR Irradiation of Human Skin

The source of UVR (290–400 nm wavelength range) was a Xenon Arc Multiport Solar Simulator 601–300 W (Solar Light Company, Philadelphia, PA, USA) equipped with a Schott-type WG320 filter to eliminate shorter wavelength UV-C, and a Schott UG11 filter to remove visible light and infrared radiation [30,31]. UVR irradiance was measured using a PMA2100 UV detector with a PMA 2108 UV-B probe (Solar Light Company).

**Figure 1:**
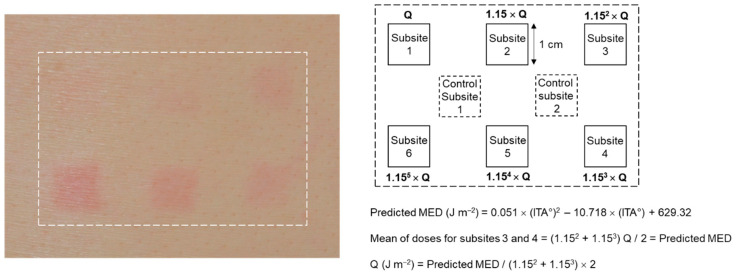
Irradiation of test skin site of a human subject with ultraviolet radiation (UVR). A typical image of the subject’s back skin site after UVR irradiation is shown. The subsites 1 to 6 were irradiated with UVR at different doses expressed as multiples of Q (J m^−2^), which was adjusted based on the ‘predicted minimal erythema dose’ (MED). The ‘predicted MED’ was calculated from the ITA° value of the subject’s back skin before irradiation. The control subsites 1 and 2 were non-irradiated.

As shown in Figure 1, a test skin site (6 × 4 cm^2^) on the back of each subject was marked and UVR was irradiated to six designated subsites. The UVR doses to be irradiated to subsites 1 to 6 were increased stepwise by 1.15 times, and the median value of the UVR doses of subsites 3 and 4 was adjusted to be equal to the ‘predicted MED’. The control subsites 1 and 2 in marginal areas served as non-irradiated controls. After UVR irradiation of the skin, the subject avoided any UVR exposure to the test skin site until visual and instrumental assessments of the MED.

### 2.4. Visual Assessment of MED

Within 16 to 24 h after UVR irradiation of the test skin site, a visual assessment of erythema was performed by an experienced examiner in a laboratory with an illumination of 450 lux. The criteria for erythema are shown in Table 1. If an adverse skin reaction of grade 1 or 2 was observed in the UVR-irradiated subsite, erythema formation was judged positive. Among the subsites with positive erythema formation, the one irradiated with the lowest UVR dose was selected, and the UVR dose irradiated to this subsite was referred to as the ‘visual MED’.

### 2.5. Instrumental Assessment of MED

The ‘instrumental MED’ of the test skin site was determined in the subjects who had undergone a visual assessment of the MED after UVR irradiation. The skin color parameters of subsites 1 to 6, which were irradiated with different doses of UVR, as well as two non-irradiated control subsites, were measured using a spectrophotometer CM-2500d. Among the skin color parameters, the a* value was utilized to estimate the degree of erythema. The a* values of the UVR-irradiated subsites were subtracted by the average value of two non-irradiated control subsites to obtain the Δa* values. The Δa*_VMED_ value was defined as the Δa* values of the subsites irradiated with UVR at the ‘visual MED’. The criterion value for the instrumental MED was obtained by averaging the Δa*_VMED_ values of all subjects. The MED was re-evaluated by applying this criterion value. The ‘instrumental MED’ was defined as the lowest dose of UVR at which the Δa* value reaches this criterion value and it was determined from the scatter plot of the Δa* values versus the UVR doses for each subject. The ‘instrumental MED’ was calculated using the first-order equation of a straight line that connects two points above and below the criterion value.

### 2.6. Statistical Analysis

The data were analyzed using the IBM SPSS Statistics version 22 software program (IBM, Chicago, IL, USA). The data are presented as the mean ± standard deviation (SD). The normality of the datasets was examined using the Kolmogorov–Smirnov test and Shapiro–Wilk test at the *p* < 0.05 level. The differences between parametric datasets were analyzed using the paired *t*-test, whereas the Wilcoxon signed-rank test was used to compare non-parametric datasets. The Pearson correlation analysis was used to test the linear association between parametric datasets at the *p* < 0.05 level. Spearman’s rank correlation analysis and Kendall’s rank correlation analysis were used to test the correlation between non-parametric datasets.

## 3. Results

### 3.1. Information on Subjects

Brief information on the human subjects and their skin characteristics are summarized in Table 2. The age of the 14 subjects ranged from 27 to 57 years (48.93 ± 8.54), and there were 13 women and 1 man. There were 3 subjects with Fitzpatrick skin type II and 11 with type III. The ITA° values of all subjects ranged from 28.2 to 46.1 (40.39 ± 4.78).

### 3.2. Visually Determined MED

The ‘predicted MED’ values for UVR calculated from the skin color parameter ITA° are shown in Table 2. Six doses of UVR selected based on the ‘predicted MED’ were irradiated to six subsites of the test skin site in each subject.

The results of the visual assessment of MED are also shown in Table 2. The UVR doses for subsites judged positive for erythema formation in the visual evaluation were underlined, and the lowest dose among these, marked with a bold font, was designated as the ‘visual MED’. The average ‘visual MED’ value of all subjects measured after UVR irradiation was 300.14 ± 84.16, whereas the average ‘predicted MED’ value calculated from the ITA° before UVR irradiation was 280.79 ± 32.78.

### 3.3. Spectrophotometric Measurement of the a* Values

The a* values of the UVR-irradiated subsites and non-irradiated control subsites were measured using a spectrophotometer, and the results are shown in Table 3. The a* values of the UVR-irradiated subsites were mostly larger than those of the non-irradiated control subsites. Also, they tended to increase with the UVR dose, although there were exceptions. The average a* value of the subsites irradiated with UVR at the ‘visual MED’ was 8.27 ± 1.22, higher than that of the non-irradiated control subsites (6.40 ± 1.03).

### 3.4. Instrumental Criterion for MED

The a* values of the UVR-irradiated subsites were corrected for that of the non-irradiated control subsites, and the resulting Δa* values are shown in Table 4. The Δa* values of the subsites irradiated with the UVR at the ‘visual MED’ were referred to as the Δa*_VMED_. The average Δa*_VMED_ value of all subjects was calculated to be 1.88 ± 0.81 in this study, and this value was used as the criterion value for the ‘instrumental MED’ of each subject. In other words, the ‘instrumental MED’ of each subject is defined as the lowest UVR dose that causes the Δa* value to be equal to the criterion value (1.88 in this study).

### 3.5. Instrumental Determination of MED

**Figure 2:**
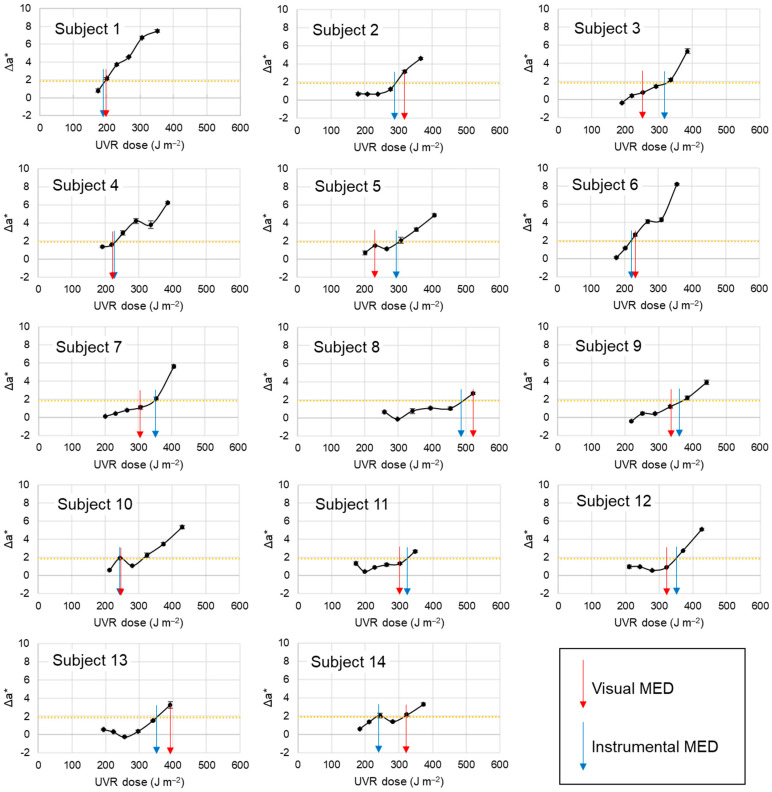
Scatter plots of the Δa* values versus UVR doses in human subjects. Data are presented as mean ± SD (*n* = 3). The criterion value (Δa* = 1.88) for the ‘instrumental MED’ for UVR is shown in a dotted yellow line. The ‘visual MED’ and ‘instrumental MED’ values are marked with red and blue arrows, respectively.

Figure 2 shows scatter plots of the Δa* values versus UVR doses in all subjects. The criterion value (Δa* = 1.88) for the ‘instrumental MED’ is shown in a dotted yellow line. A blue arrow was used to designate the ‘instrumental MED’ for UVR in each subject, at which the Δa* value reached the criterion line. The determined ‘instrumental MED’ values are comparable to the ‘visual MED’ values marked with red arrows.

### 3.6. Comparison Between the ‘Predicted MED’, ‘Visual MED’, and ‘Instrumental MED’ Values

Table 5 compares the ‘predicted MED’, ‘visual MED’, and ‘instrumental MED’ values of all subjects. The ‘visual MED’ and ‘instrumental MED’ had similar mean values (300.14 ± 84.16 and 303.29 ± 77.99, respectively) that are more different from that of the ‘predicted MED’ (280.79 ± 32.78). The normality of each MED dataset was checked by two different tests. In the Kolmogorov–Smirnov test, the ‘predicted MED’, ‘visual MED’, and ‘instrumental MED’ all showed normality, whereas in the Shapiro–Wilk test, the ‘predicted MED’ and ‘instrumental MED’ showed normality, but the ‘visual MED’ did not. Since the two tests did not give perfectly consistent results, we reserved judgment on the normality of the visual MED dataset and proceeded to the analysis of the differences between different datasets using two different tests. The paired *t*-test was applied for parametric analysis between datasets that showed normality, and the Wilcoxon signed-rank test was applied for non-parametric analysis between datasets that did not show normality. Both tests showed that there was no statistically significant difference between the means of the ‘predicted MED’, ‘visual MED’, and ‘instrumental MED’ values at *p* < 0.05.

**Figure 3:**
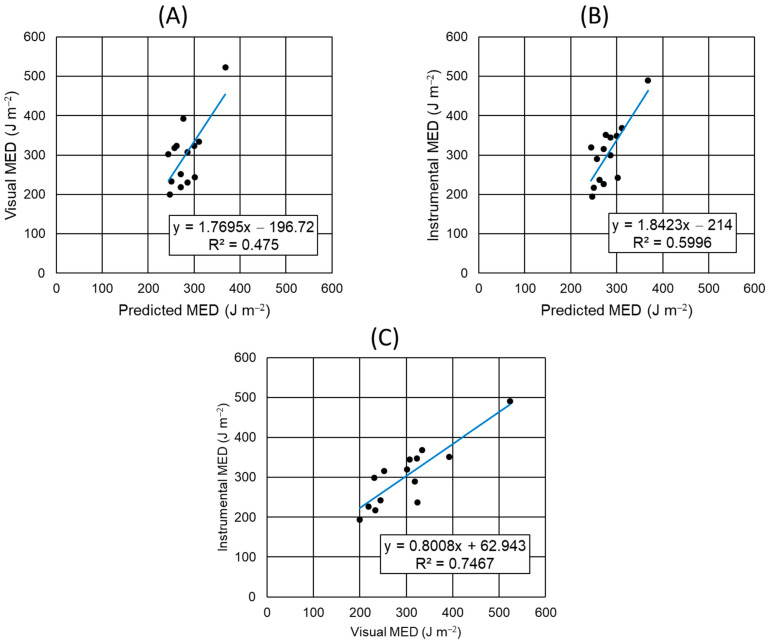
The simple linear regression analysis between the ‘predicted MED’, ‘visual MED’, and ‘instrumental MED’ values for UVR in human subjects. Scatter plots of the ‘visual MED’ versus ‘predicted MED’ (**A**), ‘instrumental MED’ versus ‘predicted MED’ (**B**), and ‘instrumental MED’ versus ‘visual MED’ values (**C**) are shown. A blue trend line, equation, and determination coefficient (R^2^) are shown in each scatter plot.

Figure 3 shows scatter plots of the ‘visual MED’ versus ‘predicted MED’, ‘instrumental MED’ versus ‘predicted MED’, and ‘instrumental MED’ versus ‘visual MED’ values from the current study. A simple linear regression analysis of each scatter plot provided a trend line and determination coefficient (R^2^). The ‘predicted MED’ values had a linear relation to the ‘visual MED’ (trend line slope = 1.7695, R^2^ = 0.4750) or the ‘instrumental MED’ values (trend line slope = 1.8423, R^2^ = 0.5996). The ‘visual MED’ and ‘instrumental MED’ values had a very high linear relation to each other (trend line slope = 0.8008, R^2^ = 0.7467).

### 3.7. Correlation Between the ‘Predicted MED’, ‘Visual MED’, and ‘Instrumental MED’ Values

Pearson correlation analysis was used to examine the linear correlations between datasets with normal distributions. The Pearson correlation coefficients (r) and *p* values between the ‘predicted MED’, ‘visual MED’, and ‘instrumental MED’ of all subjects are summarized in Table 6. The data showed that the ‘predicted MED’ values strongly correlated with the ‘visual MED’ (r = 0.689, *p* = 0.006) or the ‘instrumental MED’ values (r = 0.774, *p* = 0.001). In addition, a very strong positive correlation existed between the ‘visual MED’ and ‘instrumental MED’ values (r = 0.864, *p* = 0.000).

Meanwhile, since the normality of the ‘visual MED’ dataset could not be completely confirmed as seen above, additional non-parametric correlation analyses were performed. The results of Spearman’s rank correlation analysis and Kendall’s rank correlation analysis are shown in Table 7 and Table 8, respectively. In both tests, the ‘visual MED’ did not show a significant correlation with the ‘predicted MED’ at *p* < 0.05. However, the ‘instrumental MED’ showed a significant correlation with the ‘predicted MED’ and a strong correlation with the ‘visual MED’.

### 3.8. Modification of Equations for Predicting MED from ITA°

**Figure 4:**
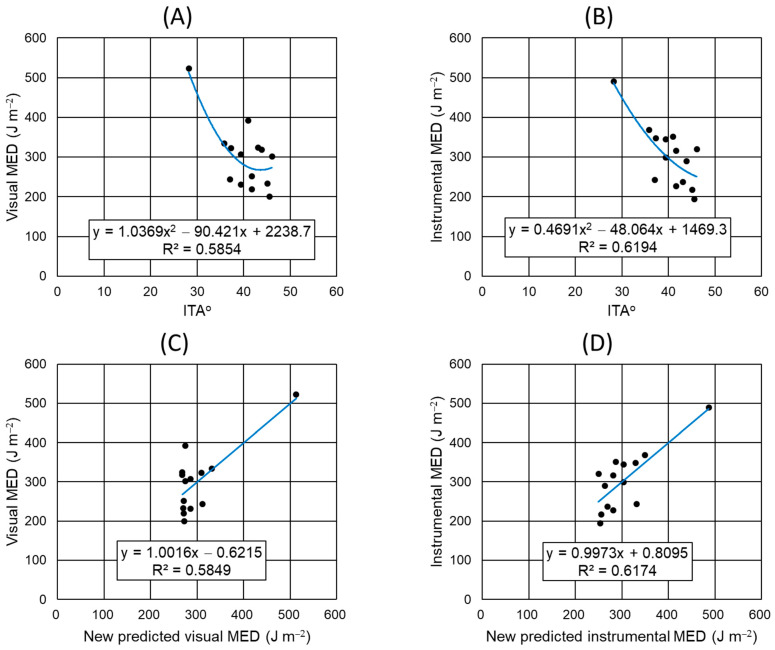
Development and validation of the new second-order equations predicting the visual and instrumental MED values from ITA° values. Scatter plots of the ‘visual MED’ versus ITA° (**A**), ‘instrumental MED’ versus ITA° (**B**), ‘visual MED’ versus ‘new predicted visual MED’ (**C**), and ‘instrumental MED’ versus ‘new predicted instrumental MED’ values (**D**) are shown. A blue trend line, equation, and determination coefficient (R^2^) are shown in each scatter plot.

The new second-order equations were derived from the relationship between the ITA° values and ‘visual MED’ or ‘instrumental MED’ values measured in this study, and are shown in Figure 4A and 4B, respectively. The new equations were then used to calculate the ‘new predicted visual MED’ or ‘new predicted instrumental MED’ for UVR. The scatter plots of the ‘visual MED’ versus ‘new predicted visual MED’ and ‘instrumental MED’ versus ‘new predicted instrumental MED’ are shown in Figure 4C and 4D, respectively. In these scatter plots, the trend lines showed slopes close to 1, demonstrating that the new equations could help predict the visual or instrumental MED values for UVR in Korean subjects.

## 4. Discussion

Various commercial products, such as sun-blocking or UVR-blocking cosmeceuticals, clothing, and glasses, are used to reduce UVR exposure levels and harmful effects [32]. The performance of various UVR protection products can be evaluated by in vitro analysis methods, but for products directly applied to the skin, it is essential to verify their efficacy under actual use conditions through human application tests [33,34]. We usually evaluate how much the test product reduces skin erythema caused by UVR and quantify how much the product increases the MED of human skin [35].

Up to now, the MED of human skin has mainly been determined visually by experts, however, this method has the limitation that the assessment of erythema varies depending on the examiner. Since the basic skin color before or after UVR irradiation varies from subject to subject, it is difficult for the examiner to apply a consistent criterion for erythema. Considering that UVR-induced erythema is characterized by changes in skin color, especially an increase in the a* value (a chrominance parameter presenting greenness to redness), we devised an instrumental evaluation method of the MED which measures the a* values with a spectrophotometer in the present study.

In a clinical trial conducted on 14 subjects, the a* values of the UVR-irradiated subsites corresponding to the ‘visual MED’ were larger than that of the non-irradiated control subsites by 1.88 ± 0.81, which was referred to as the average Δa*_VMED_ value of all subjects. This means that the examiner judged the erythema formation positive when UVR irradiation caused Δa* ≥ 1.88. We chose this value as a criterion for the ‘instrumental MED’ in the present study. When the MED was re-evaluated by applying this criterion value, all subjects’ measured ‘instrumental MED’ values were similar to the ‘visual MED’ values (303.29 ± 77.99 and 300.14 ± 84.16, respectively). Pearson correlation analysis showed a very strong positive correlation between these two variables (r = 0.864, *p* = 0.000). Spearman’s rank correlation analysis and Kendall’s rank correlation analysis also supported strong correlations between the ‘visual MED’ and ‘instrumental MED’. Thus, the instrumental evaluation method can complement or replace the visual evaluation method for MED determination.

The ∆a* = 1.88 is the result obtained in this study with a limited number of Koreans, so it is unclear whether the criterion value can be applied to all races. For now, we suggest that each laboratory develop a criterion value for each race using a similar approach. In the future, when the criterion values built by multiple laboratories can be integrated, an agreed criterion value is expected to be set and used as an international standard. Measuring differences in skin color parameters according to the presence or absence of erythema in a large population exposed to UVR and unexposed would also help establish agreed MED assessment criteria.

The results of the present study showed that the instrumental assessment of the MED is possible by measuring skin color parameters with a spectrophotometer before and after UVR irradiation or with and without UVR irradiation and analyzing the changes or differences in the skin color parameters. This instrumental evaluation method can be compared to the existing visual evaluation method in that the steps of setting the skin test area, determining the UVR irradiation dose, and irradiating the UVR are carried out in the same manner, but the reading steps of the UVR- irradiated and non-irradiated areas are carried out differently. In other words, the difference is whether the reading is conducted visually or after measuring with an instrument.

The instrumental evaluation method can be used even when there are large differences in basic skin color parameters between subjects because the differences can be corrected by subtracting the non-UVR irradiation control values from the post-UVR irradiation values of each subject. This method also has the advantage of assessing the MED more consistently than the visual evaluation method because the former compares the changes in skin color parameters of each subject with a fixed numerical criterion. The implementation of instrumental evaluation will contribute to reducing data variance between examiners and laboratories. These advantages are considered to outweigh the disadvantages of having to add instrumental measurement and data curation processes that are not included in visual evaluation methods.

The application range of the instrumental evaluation method for the MED is expected to be wide. It can be used to determine the SPFs of various commercial products including sunscreen cosmeceuticals [15,19]. It can also be used to evaluate the effects of various antioxidants and anti-inflammatory agents on the MED for UVR [36,37]. The instrumental method can be used to determine the MED for other skin inflammation inducers, such as chemicals, heat, abrasion, etc. [13,38].

Data on the basal ITA° values of the skin and MED values for UVR in human subjects have been accumulated in multiple labs worldwide, and the second-order equation has been derived to express the relationship between the ITA° and MED values [24]. The equation was used in predicting the MED values and setting the range of the UVR irradiation doses in the present study. The average of the ‘predicted MED’ values of all subjects (280.79 ± 32.78) did not statistically differ from that of the ‘instrumental MED’ or ‘visual MED’ values empirically measured after UVR irradiation. In the correlation analysis, the ‘predicted MED’ values had a strong positive correlation with the measured ‘visual MED’ (r = 0.689, *p* = 0.006) or ‘instrumental MED’ values (r = 0.774, *p* = 0.001). This result supports the usefulness of the ITA° values measured before UVR irradiation in predicting the MED for UVR.

However, since the original equation was derived by integrating data from various races, there are limitations in directly applying it to clinical trials conducted in a lab by recruiting specific races. We derived new modified second-order equations specialized for Koreans from the relationship between the ITA° values and ‘visual MED’ or ‘instrumental MED’ values measured in this study. This modified equation improved the accuracy of the MED prediction. Therefore, we believe that other individual labs can use a similar approach to derive equations specialized for their test groups and utilize them to improve the accuracy of the MED prediction.

Advanced technologies involving image analysis and artificial intelligence are being developed to replace the visual assessment of examiners in the diagnosis of various types of skin diseases accompanied by changes in shape and color [13,39]. The measurement MED is expected to be widely used because it has great clinical or industrial relevance and significance [40,41]. The present study contributed to the technical improvement of the instrumental method for measuring the MED. On the other hand, there are several limitations to the instrumental evaluation method developed in this study to be proposed as a global standard. Since there may be differences in results due to various factors such as the subject’s race, measured skin area, skin characteristics, UVR irradiation device and method, and skin color measurement device, it is recommended that each lab prepare and use its own protocol according to the approach presented in this study before an internationally accepted standard method is established.

## 5. Conclusions

This study introduced an instrumental evaluation method to determine the MED of each human subject based on the spectrophotometric measurement of the skin color parameter a*. The instrumental evaluation method has the advantage of avoiding the variation and errors that can arise from the subjective visual evaluation method and enabling more consistent and quantitative evaluations based on numerical data.

## Figures and Tables

**Table 1:** Criteria for visual assessment of erythema after UVR irradiation of human skin.

Grade of Skin Reaction	Description	Erythema Assessment
0	No observable adverse reaction of the irradiated skin area.	Negative
0.5	Erythema occurs in less than 50% of the skin area irradiated with UVR or the erythema has unclear boundaries.	Negative
1	Erythema with clear boundaries appears in more than 50% of the skin area irradiated with UVR.	Positive
2	The full skin area irradiated with UVR shows a clear erythema reaction and the skin surface is swollen.	Positive

**Table 2:** Subjects’ information and visual assessment of MED for UVR.

Subject No.	Age	Gender	Skin Characteristic		‘Predicted MED’ (J m^−2^)	Irradiated UVR Doses (J m^−2^)						‘Visual MED’ (J m^−2^)
			Fitzpatrick Skin Type	ITA°		Subsite 1	Subsite 2	Subsite 3	Subsite 4	Subsite 5	Subsite 6	
1	57	F	II	45.6	247	174	**200**	230	266	305	351	200
2	53	F	III	43.9	257	181	208	239	276	**318**	365	318
3	51	F	III	41.7	271	191	219	**252**	291	335	385	252
4	39	F	III	41.7	271	191	**219**	252	291	335	385	219
5	54	F	III	39.4	286	201	**231**	266	307	354	407	231
6	55	F	III	45.1	250	176	202	**233**	269	309	355	233
7	27	F	II	39.4	286	201	231	266	**307**	354	407	307
8	46	F	III	28.2	368	259	298	342	396	455	**523**	523
9	56	F	III	35.8	311	219	252	289	**334**	384	442	334
10	55	F	III	37.1	302	212	**244**	281	325	373	429	244
11	43	F	II	46.1	244	172	197	227	262	**302**	347	302
12	53	M	III	37.3	300	211	243	279	**323**	371	427	323
13	42	F	III	41.0	276	194	223	257	297	341	**392**	392
14	54	F	III	43.1	262	184	212	244	282	**324**	372	324
Mean	48.93			40.39	280.79							300.14
SD	8.54			4.78	32.78							84.16

The UVR doses for the subsites judged positive for erythema formation by the visual examiner are underlined. The lowest dose among these in each subject is designated as the ‘visual MED’ and marked with a bold font. SD, standard deviation.

**Table 3:** The a* values at the UVR-irradiated subsites and the non-irradiated control subsites in human subjects.

Subject No.	a* Values									
	Control Subsite 1	Control Subsite 2	Mean of Controls	Subsite 1	Subsite 2	Subsite 3	Subsite 4	Subsite 5	Subsite 6	Subsites with ‘Visual MED’
1	5.72	5.24	5.48	6.29	**7.65**	9.20	10.05	12.21	12.98	7.65
2	4.89	4.15	4.52	5.22	5.19	5.20	5.73	**7.69**	9.13	7.69
3	5.80	6.22	6.01	5.65	6.45	**6.80**	7.49	8.20	11.39	6.80
4	5.45	5.91	5.68	7.02	**7.25**	8.54	9.87	9.46	11.89	7.25
5	5.85	5.70	5.78	6.47	**7.24**	6.92	7.84	9.07	10.64	7.24
6	5.52	5.51	5.52	5.64	6.68	**8.15**	9.61	9.82	13.74	8.15
7	7.41	7.51	7.46	7.58	7.90	8.27	**8.56**	9.53	13.10	8.56
8	8.10	8.04	8.07	8.71	7.92	8.81	9.13	9.10	**10.76**	10.76
9	6.40	6.66	6.53	6.12	6.98	6.95	**7.74**	8.69	10.42	7.74
10	8.05	8.10	8.08	8.69	**10.02**	9.17	10.34	11.58	13.45	10.02
11	5.87	5.92	5.90	7.25	6.37	6.81	7.11	**7.23**	8.57	7.23
12	7.30	6.37	6.84	7.83	7.82	7.42	**7.75**	9.60	11.97	7.75
13	6.62	6.74	6.68	7.26	7.01	6.44	7.06	8.24	**9.92**	9.92
14	6.66	7.30	6.98	7.56	8.32	9.01	8.36	**9.08**	10.24	9.08
Mean			6.40							8.27
SD			1.03							1.22

The UVR doses irradiated to the subsites in each subject are shown in Table 2. The subsites judged positive for erythema formation by the visual examiner are underlined. Among these, the one irradiated with the lowest dose of UVR is marked with a bold font.

**Table 4:** The Δa* values at the UVR-irradiated subsites in human subjects.

Subject No.	Δa* Values						
	Subsite 1	Subsite 2	Subsite 3	Subsite 4	Subsite 5	Subsite 6	Subsites with ‘Visual MED’
1	0.81	**2.17**	3.72	4.57	6.73	7.50	2.17
2	0.7	0.67	0.68	1.21	**3.17**	4.61	3.17
3	−0.36	0.44	**0.79**	1.48	2.19	5.38	0.79
4	1.34	**1.57**	2.86	4.19	3.78	6.21	1.57
5	0.69	**1.46**	1.14	2.06	3.29	4.86	1.46
6	0.12	1.16	**2.63**	4.09	4.30	8.22	2.63
7	0.12	0.44	0.81	**1.10**	2.07	5.64	1.10
8	0.64	−0.15	0.74	1.06	1.03	**2.69**	2.69
9	−0.41	0.45	0.42	**1.21**	2.16	3.89	1.21
10	0.61	**1.94**	1.09	2.26	3.5	5.37	1.94
11	1.35	0.47	0.91	1.21	**1.33**	2.67	1.33
12	0.99	0.98	0.58	**0.91**	2.76	5.13	0.91
13	0.58	0.33	−0.24	0.38	1.56	**3.24**	3.24
14	0.58	1.34	2.03	1.38	**2.10**	3.26	2.10
Mean							1.88
SD							0.81

The UVR doses irradiated to the subsites in each subject are shown in Table 2. The subsites judged positive for erythema formation by the visual examiner are underlined. Among these, the one irradiated with the lowest dose of UVR is marked with a bold font. To estimate the Δa* values for the UVR-irradiated subsites, their a* values were subtracted by the average value of two non-irradiated control subsites shown in Table 3.

**Table 5:** The ‘predicted MED’ values and the measured ‘visual MED’ and ‘instrumental MED’ values for UVR in human subjects.

Subject No.	‘Predicted MED’ (J m^−2^)	‘Visual MED’ (J m^−2^)	‘Instrumental MED’ (J m^−2^)
1	247	200	194
2	257	318	290
3	271	252	316
4	271	219	227
5	286	231	299
6	250	233	217
7	286	307	345
8	368	523	490
9	311	334	369
10	302	244	243
11	244	302	320
12	300	323	348
13	276	392	351
14	262	324	237
Mean	280.79	300.14	303.29
SD	32.78	84.16	77.99
Kolmogorov–Smirnov test *p*-value	0.200 *	0.131	0.200 *
Shapiro–Wilk test *p*-value	0.064	0.046 Data normality rejected	0.335
Paired *t*-test *p*-value	0.292 vs. ‘visual MED’; 0.160 vs. ‘instrumental MED’	0.787 vs. ‘instrumental MED’	
Wilcoxon signed-rank test *p*-value	0.233 vs. ‘visual MED’; 0.140 vs. ‘instrumental MED’	0.730 vs. ‘instrumental MED’	

* This is a lower bound of the true significance.

**Table 6:** Pearson correlation analysis between the ‘predicted MED’, ‘visual MED’, and ‘instrumental MED’ values for UVR in human subjects.

Variables	‘Predicted MED’		‘Visual MED’	
	r	*p*	r	*p*
‘Visual MED’	0.689	0.006		
	A strong positive correlation			
‘Instrumental MED’	0.774	0.001	0.864	0.000
	A strong positive correlation		A very strong positive correlation	

Pearson correlation coefficients (r) and *p* values are shown. The correlation is considered statistically significant when *p* < 0.05. The absolute values of r indicate a very weak (0 < |r| < 0.2), weak (0.2 **≤** |r| < 0.4), moderate (0.4 ≤ |r| < 0.6), strong (0.6 ≤ |r| < 0.8), or very strong (0.8 ≤ |r| < 1) correlation between the two variables. The plus or minus sign of the r value indicates a positive or negative correlation between them.

**Table 7:** Spearman’s rank correlation analysis between the ‘predicted MED’, ‘visual MED’, and ‘instrumental MED’ values for UVR in human subjects.

Variables	‘Predicted MED’		‘Visual MED’	
	Spearman’s Rank Correlation Coefficient Rho (ρ)	*p*-Value	Spearman’s Rank Correlation Coefficient Rho (ρ)	*p*-Value
‘Visual MED’	0.471	0.089		
	Not significant			
‘Instrumental MED’	0.654	0.011	0.793	0.001
	A strong positive correlation		A strong positive correlation	

**Table 8:** Kendall’s rank correlation analysis between the ‘predicted MED’, ‘visual MED’, and ‘instrumental MED’ values for UVR in human subjects.

Variables	‘Predicted MED’		‘Visual MED’	
	Kendall’s Rank Correlation Coefficient Tau (τ)	*p*-Value	Kendall’s Tau (τ)	*p*-Value
‘Visual MED’	0.367	0.070		
	Not significant			
‘Instrumental MED’	0.522	0.010	0.670	0.001
	A moderate positive correlation		A strong positive correlation	

## Data Availability

Data may be available from Jae Sook Koh upon request due to ethical reasons.

## Abbreviations

ITA°	individual typology angle
MED	minimal erythema dose
ROS	reactive oxygen species
UVR	ultraviolet radiation
SD	standard deviation
SPF	sun protection factor
